# Ethanol potentiates the genotoxicity of the food-derived mammary carcinogen PhIP in human estrogen receptor-positive mammary cells: mechanistic support for lifestyle factors (cooked red meat and ethanol) associated with mammary cancer

**DOI:** 10.1007/s00204-018-2160-9

**Published:** 2018-01-23

**Authors:** Durr-e-shahwar Malik, Rhiannon M. David, Nigel J. Gooderham

**Affiliations:** 1 Computational and Systems Medicine, Imperial College London, London, SW7 2AZ UK; 2 Present Address: Discovery Safety and Metabolism, AstraZeneca, Cambridge, CB4 0WG UK

**Keywords:** 2-amino-1-methyl-6-phenylimidazo[4,5-b]pyridine, Ethanol, Estrogen receptor, Mammary carcinogen, Genotoxicity, CYP

## Abstract

Consumption of cooked/processed meat and ethanol are lifestyle risk factors in the aetiology of breast cancer. Cooking meat generates heterocyclic amines such as 2-amino-1-methyl-6-phenylimidazo[4,5-b]pyridine (PhIP). Epidemiology, mechanistic and animal studies indicate that PhIP is a mammary carcinogen that could be causally linked to breast cancer incidence; PhIP is DNA damaging, mutagenic and oestrogenic. PhIP toxicity involves cytochrome P450 (CYP1 family)-mediated metabolic activation to DNA-damaging species, and transcriptional responses through Aryl hydrocarbon receptor (AhR) and estrogen-receptor-α (ER-α). Ethanol consumption is a modifiable lifestyle factor strongly associated with breast cancer risk. Ethanol toxicity involves alcohol dehydrogenase metabolism to reactive acetaldehyde, and is also a substrate for CYP2E1, which when uncoupled generates reactive oxygen species (ROS) and DNA damage. Here, using human mammary cells that differ in estrogen-receptor status, we explore genotoxicity of PhIP and ethanol and mechanisms behind this toxicity. Treatment with PhIP (10^−7^–10^−4^ M) significantly induced genotoxicity (micronuclei formation) preferentially in ER-α positive human mammary cell lines (MCF-7, ER-α+) compared to MDA-MB-231 (ER-α−) cells. PhIP-induced CYP1A2 in both cell lines but CYP1B1 was selectively induced in ER-α(+) cells. ER-α inhibition in MCF-7 cells attenuated PhIP-mediated micronuclei formation and CYP1B1 induction. PhIP-induced CYP2E1 and ROS via ER-α-STAT-3 pathway, but only in ER-α (+) MCF-7 cells. Importantly, simultaneous treatments of physiological concentrations ethanol (10^−3^–10^−1^ M) with PhIP (10^−7^–10^−4^ M) increased oxidative stress and genotoxicity in MCF-7 cells, compared to the individual chemicals. Collectively, these data offer a mechanistic basis for the increased risk of breast cancer associated with dietary cooked meat and ethanol lifestyle choices.

## Introduction

Epidemiology studies show that lifestyle choices such as consumption of red or processed meat and ethanol are consistently associated with the development of cancers of the gastrointestinal tract, the mammary gland and the prostate (Abid et al. 2014; Boada et al. 2016; Cross et al. 2007; Major et al. 2011; Sinha et al. 2009; Willett 1994). This and supporting experimental data has prompted IARC to declare red meat as a Class 2A human carcinogen (probably carcinogenic in humans) and processed meat as a Class 1 carcinogen (carcinogenic in humans) (https://www.iarc.fr/en/media-centre/pr/2015/pdfs/pr240_E.pdf). The cooking of red and processed meat generates chemicals that are DNA damaging, mainly polycyclic aromatic hydrocarbons and heterocyclic amines (David et al. 2016; Felton et al. 1991; Holland et al. 2004; Sinha et al. 2005). 2-Amino-1-methyl-6-phenylimidazo[4,5-b]pyridine (PhIP) is one of the most abundant heterocyclic amines present in well-done meat (Gooderham et al. 1997) (Zheng and Lee 2009). PhIP is a genotoxic carcinogen that is metabolically activated by CYP1 family enzymes (particularly CYP1A2) to *N*-hydroxy-PhIP (Boobis et al. 1994; Zhao et al. 1994). Subsequent esterification of the *N*-hydroxy metabolite produces unstable products that spontaneously form nitrenium ions and attack DNA to induce mutation (Gooderham et al. 2007; Langouet et al. 2002; Lynch et al. 1998; Turesky et al. 1998, 2002; Yadollahi-Farsani et al. 1996; Zhu et al. 2000). PhIP has been shown to induce tumours in rats in hormonally responsive tissues (breast, prostate, colon) (Ito et al. 1991; Shirai et al. 1997) and is reported by IARC (https://monographs.iarc.fr/ENG/Monographs/vol56/mono56-13.pdf, 1993) to be a Class 2B carcinogen (possibly carcinogenic to humans). In addition to being a genotoxic carcinogen, an important characteristic of PhIP is its hormone-like activity (estrogen-like) (Lauber et al. 2004; Papaioannou et al. 2014). Estrogens, known to promote breast carcinogenesis (Liehr 2001), primarily act *via* two receptors, estrogen-receptor α (ER-α) and ER-β with ER-α being more abundantly expressed (Hewitt and Korach 2003) in approximately two-thirds of breast tumors and its presence determines the responsiveness towards hormone therapy (Williams et al. 2008). Interestingly, PhIP exhibits its estrogenic activity exclusively *via* ER-α (Lauber et al. 2004). The estrogenic behavior of PhIP has been shown to increase the invasiveness of breast cancer cells (Lauber and Gooderham 2011) but the role of ER in the genotoxicity and metabolic activation of PhIP has not been explored.

A number of cytochrome P450 enzymes (CYPs) are known to be involved in metabolism of steroid hormones, particularly CYP1A1, 1A2 and 1B1 (Go et al. 2015). Additionally, CYP2E1 is reported to be differentially expressed in hormone-responsive MCF-7 cells compared to non-responsive MDA-MB-231 cells (Leung et al. 2013). Moreover, female steroid hormones (estrogen and progesterone) are known to regulate CYP2E1 expression (Konstandi et al. 2013). In view of the regulation of CYP2E1 *via* estrogen and the hormone-like activity (estrogen) of PhIP (Lauber and Gooderham 2007), the possibility exists that PhIP might regulate CYP2E1 expression.

Epidemiology shows that consumption of ethanol is associated with breast cancer (Hamajima et al. 2002; Singletary and Gapstur 2001; Smith-Warner et al. 1998), with an intake of 10 g ethanol per day (approximately 1.25 units) increasing the risk of breast cancer between 6–10% (IARC 2012 https://monographs.iarc.fr/ENG/Monographs/vol96/mono96.pdf). Social consumption of ethanol readily achieves mM plasma concentrations. The risk is dose-dependent and the evidence that alcoholic drinks are a cause of pre- and post-menopausal breast cancer is sufficiently convincing that IARC have classed ethanol as a class 1 carcinogen (carcinogenic in humans) (https://monographs.iarc.fr/ENG/Monographs/vol96/mono96.pdf). Although ethanol can be metabolised to acetaldehyde, which forms adducts with DNA (Abraham et al. 2011), overall the case for ethanol being a genotoxic carcinogen is weak (https://www.gov.uk/government/publications/consumption-of-alcoholic-beverages-and-risk-of-cancer), and a non-genotoxic mode of action is likely to contribute. Thus, although epidemiological evidence supports a positive association between alcohol intake and the risk for breast cancer, a mechanistic understanding of this association is lacking.

In the present work, we describe mechanistic studies that explore the toxicity of PhIP and ethanol and their respective abilities to damage DNA. We further show the involvement of ER-α and that ethanol can potentiate the genotoxicity of the mammary carcinogen PhIP through mutually interactive biochemistry.

## Methods

### Cell culture and treatment

The human breast adenocarcinoma MCF-7 (ER-α+) and MDA-MB-231 (ER-α−) cell lines were purchased from ATCC (LGC Prochem, Middlesex,UK) and were grown in minimum essential medium (MEM) (GIBO, Life technologies, Paisley, UK) supplemented with 10% fetal bovine serum (FBS), 100 units/ml of penicillin and streptomycin 100 µg and 2 mM L-glutamine. Cells were cultured routinely in 75-cm^2^ flasks in a humidified incubator at 37 °C, 5% CO_2_. Prior to treatment, cells (MCF-7 and MDA-MB-231) at a density of 25,000 cells/well in 24-well plates, were cultured in MEM supplemented with 5% dextran-coated charcoal-stripped FBS (Stripped media) for 72 h. Cells were treated with PhIP (0–100 µM, Toronto Research Chemicals Inc., Toronto, Canada) and Estradiol (E_2_) dissolved in dimethyl sulphoxide (DMSO). For treatment with estrogen-receptor inhibitor, cells were co-treated with PhIP and selective estrogen inhibitor Fulvestrant ICI 182,780 (ICI) (Sigma-Aldrich) for 24 h. PhIP, E_2_ and ICI were dissolved in DMSO.

For STAT3 inhibition, cells were co-treated for 24 h with PhIP and 25 µM STAT3 inhibitor (STAT3 inhibitor VIII 5, 15 diphenylporphyrin, Millipore, Feltham, UK). STAT3 inhibitor was dissolved in DMSO. For ethanol treatment, media was supplemented with different concentrations of ethanol (10 mM-100 mM, Sigma-Aldrich) and was added to the cells. In some experiments, *N*-acetyl-cysteine (NAC) (10 mM, Sigma-Aldrich) diluted in phosphate-buffered saline (PBS) was added to incubations.

### Cytotoxicity and micronucleus assay

Cytotoxicity and micronucleus (MN) assays were performed according to OECD guidelines adapted to MCF-7 and MDA-MB-231 cells. Briefly, cells were seeded at a density of 5 × 10^4^ cells per well in 24-well plate. Cells were treated with PhIP or ethanol as detailed previously. Following treatment with chemicals and harvesting (48 h), cells were trypsinised, the cell concentration adjusted to 2 × 10^5^ and re-suspended in serum-free R_0_ (serum-free media) with 2% pluronic acid medium (GIBCO, Life technologies) and cytotoxicity was determined by counting cells in a haemocytometer with TrypanBlue exclusion (GIBCO, Life technologies). For the MN assay, cells were spread on a microscope slide using a cytospin. Cells at a density of 2 × 10^4^ cells per slide were fixed with 100% methanol and stained for 60 s with acridine orange (0.1 mg ml^−1^ dissolved in PBS, Sigma-Aldrich). Frequency of MN was scored in 2000 cells per sample and three biological replicates were performed per treatment. Etoposide (1.25 µM) was used as a positive control.

### Reverse transcription quantitative polymerase chain reaction (RT-qPCR)

Following treatment, cells were lysed using TRIZOL reagent and chloroform (0.2 ml) was added in each sample and centrifuged 12,000x*g* (10 min 2–8 °C). The upper aqueous phase was transferred to a fresh tube and 5 µg of RNase-free glycogen (as carrier to aqueous phase) and 0.5 ml of isopropyl alcohol was added to precipitate RNA and incubated (37 °C, 10 min). Following incubation, lysates were centrifuged at 12,000x*g* (10 min 2–8 °C). The gel-like pellet was washed with ethanol and re-dissolved in RNase-free water with heating (55–60 °C). Extracted RNA was quantified by UV spectroscopy (UV–VIS Nano-spectrophotometer, Implen, Essex, UK) and purity was assessed from 260/280 nm and 260/230 nm ratios. Reverse transcription (RT) of extracted RNA (100–500 ng) was completed according to manufacturer’s protocol (Invitrogen) and qPCR was performed using predesigned Taqman gene expression assays and FAST PCR master mix (Taqman, Applied Biosystems, Life technologies) using a StepOnePlus fast real-time PCR system (Applied Biosystems, Life technologies). Target gene expression was normalized to GAPDH and quantified using the delta-Ct method (Livak and Schmittgen 2001).

### ROS assay

Production of reactive oxygen species (ROS) was measured using 2′,7′–dichlorofluorescein diacetate method. Carboxy-2′,7′-dichlorofluorescein diacetate (carboxy-DCF-DA) is taken up by viable cells and cleaved by endogenous esterases to the nonfluorescent derivative, reduced carboxy-dichlorofluorescein (carboxy-DCFH). This product is retained in the cytosol where it can be oxidized by intracellular ROS to the highly fluorescent product, oxidised carboxy-dichlorofluorescein (carboxy-DCF). The intensity of fluorescence is proportional to intracellular ROS levels. MCF-7 cells (2 × 10^4^/well) were seeded into 24-well plates in 1% FBS and allowed to attach overnight. Cells were treated with PhIP for 24 h and then treated with Carboxy-DCFDA (20 µL, 30 µM) in media containing 1% FBS (1 ml) and incubated for 30 min at 37 °C, washed with PBS and new media (1% FBS) was added. Fluorescent measurements (excitation at 485 nm and emission at 520 nm) were then taken from 10 min to 24 h using a fluorescence plate reader (BMG POLARstar Galaxy Labtech, Ortenberg, Germany). The concentration of DMSO (0.2%) was identical in all treatments and had no effect on ROS production at this concentration.

### Statistical analysis

The difference in treatments vs. control was compared by one-way analysis of variance (ANOVA) followed by a Dunnett’s post test. Data were obtained from measurements made in at least three independent cultures and presented as a mean ± standard error (SEM). Pearson’s correlation coefficient test was used for correlation analysis (GraphPad Prism 5, GraphPad Software Inc., La Jolla, CA, USA).

## Results

### Does estrogen-receptor modulate PhIP genotoxicity?

The genotoxicity of PhIP is well established (Boyce et al. 2014; Brooks et al. 1994; Lynch et al. 1998; Yadollahi-Farsani et al. 1996). In the present study, we have assessed genotoxicity using an adapted micronucleus assay and show that PhIP is a potent inducer of micronuclei in ER receptor-positive MCF-7 cells but not MDA-MB-231 cells (Table 1). Compared to control, the highest concentration of PhIP (100 µM) showed approximately a tenfold induction in MN formation in MCF-7 cells and threefold in MDA-MB-231 cells (Table 1). Since ER-α is expressed in MCF-7 cells (but not MDA-MB-231 cells), while ER-β is weakly expressed in both cell lines (Vladusic et al. 2000), this suggests a possible role for ER-α in the increased genotoxicity of PhIP in MCF-7 cells compared to MDA-MB-231 cells. To examine the role of ER receptor, we investigated the genotoxicity of PhIP after blocking the ER-α-receptor using the potent and selective ER receptor antagonist ICI 182,780 (Bender and Veney 2008).

**Table 1:** The cytotoxicity and genotoxicity (micronucleus formation) of PhIP-treated MCF-7 and MDA-MB-231 mammary cells

	MCF-7 cells		MDA-MB-231 cells	
	Cytotoxicity^a^	MN frequency^b^	Cytotoxicity^a^	MN frequency^b^
Control	89% ± 1	3.5 ± 0.5	88.9% ± 7.2	3.5 ± 1
PhIP 100 nM	86% ± 2.4	12.8 ± 5.1	87.2% ± 0.9	3.2 ± 2.5
PhIP 1 µM	85.6% ± 2.4	22.3 ± 2.6	88.1% ± 0.9	4.7 ± 1.8
PhIP 10 µM	85.8% ± 2.6	29.2 ± 3.4	90.4% ± 4.3	9 ± 0.9
PhIP 100 µM	82.7% ± 1.7	49 ± 3.5	87.7% ± 1.5	13.7 ± 1.3
Etoposide	81.7% ±1.4	172 ± 4.8	83.7% ± 2.3	121 ± 27.7

^a^Percent survival ± Standard Deviation (SD), mean of three independent cultures, two slides per culture

^b^Micronucleus (MN) frequency presented as MN/1000 cells, mean of three independent cultures ± SD, two slides per culture, 1000 cells/slide counted

**Fig. 1:**
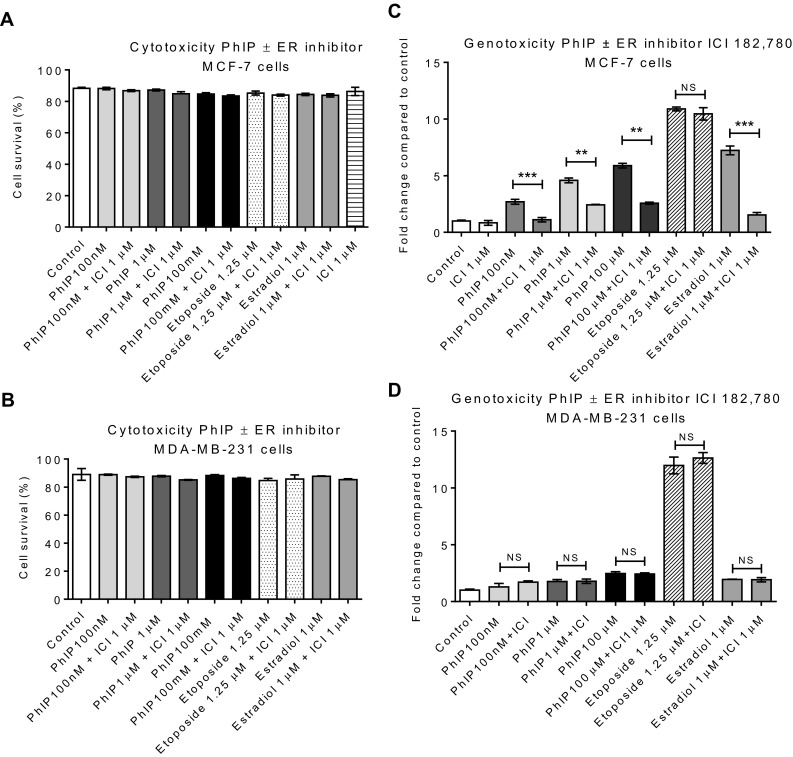
Effect of estrogen-receptor antagonist ICI, 182, 780 on the genotoxicity of PhIP in breast cells: Cells were harvested 48 h post treatment (24 h). Cytotoxicity is expressed as % cell survival as measured by cell counting using haemocytometer (**a, b**). Genotoxicity of PhIP measured by micronucleus (MN) frequency in presence/absence of ICI 182,780 in MCF-7 (**c**) and MDA-MB-231 (**d**) cells. Etoposide was used as a positive control. MN frequency per 1000 cells was measured following treatment (1000/slide and two slides per culture). Statistically significant differences between PhIP vs. PhIP & ICI 182, 780 co-treated samples were assessed by Student’s *t* test in GraphPad Prism 6. Significance is shown in *p* values; ***p < 0.001, **p < 0.01, *p < 0.05, *NS* no significant difference. Error bars represent the standard error of the mean (SEM) for independent cultures (*n* = 3)

No significant change in the cell survival was observed following treatments with PhIP, etoposide (positive control) or estradiol (Fig. 1a, b). Co-treatment with ICI significantly attenuated (*p* < 0.001) MN formation following treatment with PhIP in MCF-7 (Fig. 1c), while in MDA-MB-231, no change in the DNA damaging ability of PhIP in the presence of ER antagonist was observed (Fig. 1d). These data support a role of ER-α in the increased genotoxicity of PhIP and suggest that ER-β does not play a role. MN induction by etoposide, the positive control, was not affected by co-incubation with ICI (Fig. 1). In contrast, E_2_-mediated genotoxicity was attenuated with ICI in MCF-7 but not MDA-MB-231 cells (Fig. 1). Overall, the genotoxicity data suggest that ER-α can regulate the genotoxicity of both PhIP and E2.

### Role of ER receptor in the metabolic activation of PhIP

**Fig. 2:**
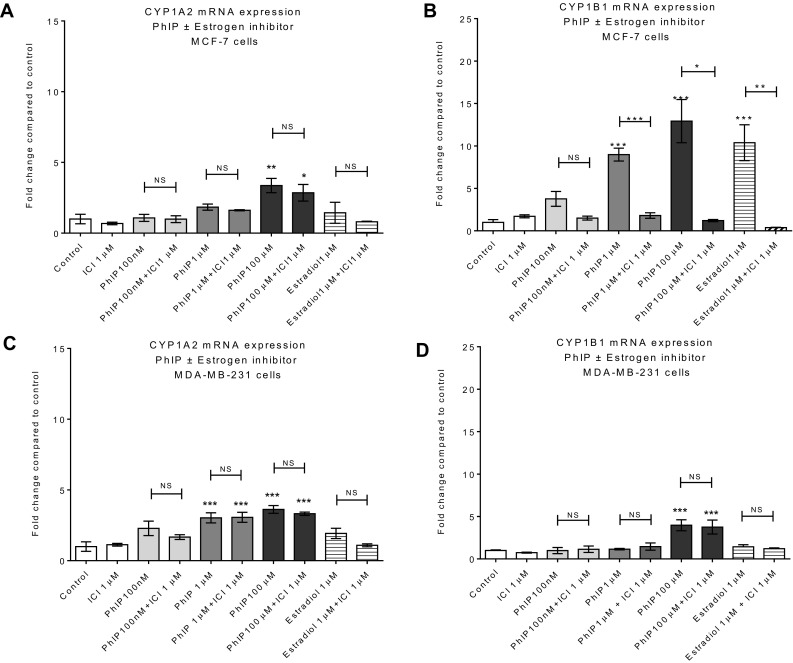
Induction of CYP1A2 and CYP1B1 mRNA expression by PhIP; in MCF-7 (**a, b**) and MDA-MB-231 (**c, d**) cells measured by RT-qPCR. Data were normalized to expression of GAPDH and are shown relative to control (0.2% DMSO). Statistically significant differences between PhIP vs. PhIP & ICI 182, 780 co-treated samples were assessed by Student’s *t* test and between control (0.2% DMSO) and treated samples using one-way ANOVA with a Dunnett’s post test in GraphPad Prism 6. Significance is shown in *p* values; ***p < 0.001, **p < 0.01, *p < 0.05, *NS* no significant difference. Error bars represent the standard error of the mean (SEM) for independent cultures (*n* = 3)

The genotoxicity of PhIP is dependent upon CYP1 family-mediated metabolic activation to *N*-hydroxy PhIP (Boobis et al. 1994; Zhao et al. 1994). This metabolite can be esterified by sulphotransferase to generate metabolites that readily form nitrenium derivatives capable of attacking DNA (Buonarati and Felton 1990; Chevereau et al. 2017; Langouet et al. 2002; Turesky et al. 1998, 2002). Thus, we investigated whether PhIP can induce gene expression of these enzymes. The results show that PhIP can induce CYP1A2/1B1 in both cell lines but that CYP1B1 induction in ER-α (+) MCF-7 was higher compared to ER-α (−) MDA-MB-231 cells (Fig. 2). PhIP failed to induce CYP1A1 in either cell line (data not shown). To further examine the role of ER-α in the regulation of CYP1B1 induction, both cell lines were co-treated with PhIP and ER inhibitor ICI for 24 h and CYP1B1 mRNA expression was determined by qPCR. E_2_ (1 µM) was used as a positive control, being the endogenous ligand for the ER receptor. Interestingly, E_2_ upregulated CY1B1 expression in ER-α (+) MCF-7 cell line only and this increase was attenuated significantly by co-treatment with ER inhibitor ICI, suggesting the possible role of ER-α in the regulation of CYP1B1 mRNA (Fig. 2b). No significant change compared to vehicle control in CYP1A2 mRNA expression was observed by E_2_ treatments in either cell line, implying the role of E_2_ in the regulation of CYP1B1 only. Overall, this suggests that E_2_ regulates CYP1B1 expression via ER-α receptor. PhIP-mediated induction of CYP1B1 was significantly inhibited (p = 0.05) by concurrent treatment with ICI (Fig. 2B) in MCF-7 cells only, indicating that PhIP can induce CYP1B1 *via* ER-α in ER-α (+) MCF-7 cells. Upregulation of CYP1A2 mRNA expression by PhIP was unaffected by ICI treatment in both cell lines (Fig. 2a, c). Overall, PhIP can upregulate CYP1A2 in both cell lines, probably via AhR, while its effects on CYP1B1 are ER-α mediated. Collectively, these multiple CYP induction mechanisms employed by PhIP can potentiate PhIP genotoxicity in ER-α (+) mammary cells.

### CYP2E1 induction by PhIP

**Fig. 3:**
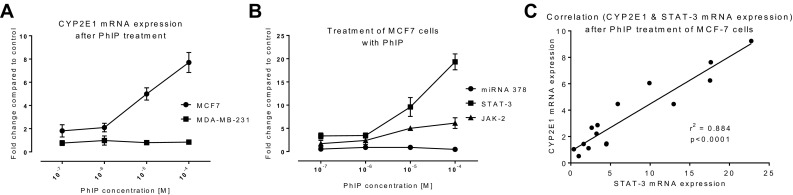
CYP2E1 mRNA expression mediated by PhIP in breast cells. CYP2E1 expression in MCF-7 and MDA-MB-231 cells (**a**) was measured by RT-qPCR. The involvement of miR378 and JAK/STAT3 pathway in MCF-7 cells treated with different concentrations of PhIP was assessed by qPCR (**b**). Data were normalized to expression of GAPDH and are shown relative to control 0.1% DMSO. Correlation of mRNA expression of CYP2E1 and STAT3 in PhIP-treated MCF-7 cells is shown in (**c**). Statistical significance was calculated by linear regression in GraphPad Prism 6, ***p < 0.001. Error bars represent SEM for independent cultures (*n* = 3)

Previous reports indicating that estrogen can regulate CYP2E1 (Konstandi et al. 2013) and the hormone-like activity (estrogenic) of PhIP (Lauber et al. 2004; Lauber and Gooderham 2007, 2011) suggest that PhIP might regulate CYP2E1, thus we investigated this in the current study. The results show that PhIP treatment induced CYP2E1 mRNA expression in a dose-dependent manner in hormone-responsive MCF-7 cells (but not in MDA-MB-231 cells) (Fig. 3a).

Recently, our laboratory has shown that transcription of STAT3 (Signal transducer and activator of transcription 3) is involved in the upregulation of CYP2E1 expression (Patel et al. 2014). Others have reported that miR378 can regulate CYP2E1 *via* translational repression (Mohri et al. 2010). To examine the roles of these mediators, we looked at the expression of miR378 and STAT3 mRNA in PhIP-treated MCF-7 cells (PhIP increased CYP2E1 expression). Following 24 h PhIP treatment, no change in miR378 expression was observed (Fig. 3b), whereas a dose-dependent upregulation of STAT3 mRNA expression was seen (Fig. 3b). This implies that miR378 has no role in PhIP-mediated CYP2E1 upregulation in MCF-7 cells. In contrast, a statistically significant (*p* ≤ 0.0001) correlation was found between STAT3 and CYP2E1 mRNA expression (Fig. 3c). As STAT3 is activated by Janus kinase 2 (JAK2), JAK2 mRNA expression was determined in PhIP-treated MCF-7 cells and a dose-dependent increase was seen (Fig. 3b). This suggests that PhIP can activate JAK/STAT3 pathway in the ER-α positive MCF-7 cells.

**Fig. 4:**
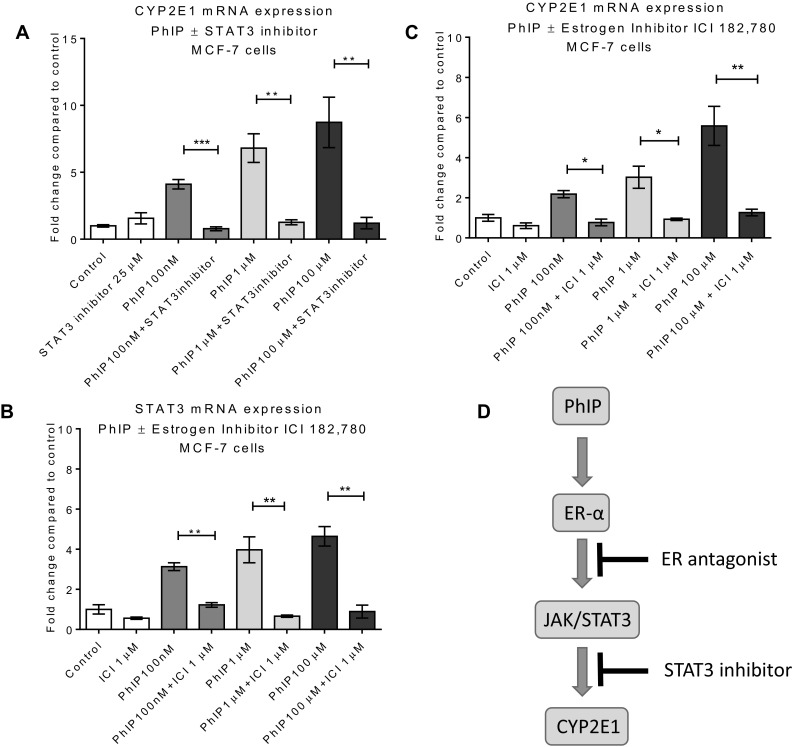
Role of estrogen receptor (ER) and STAT3 in CYP2E1 induction by PhIP in MCF-7 cells: CYP2E1 mRNA expression in the presence of STAT3 inhibitor 25 µM (**a**), STAT3 mRNA expression in the presence of PhIP and ICI 182,780 in MCF7 cells (**b**), CYP2E1 expression in the presence of ER inhibitor ICI 182,780 (1 µM) (**c**). DMSO (0.2%) was used as vehicle control. Statistically significant differences between PhIP vs. PhIP & ICI 182, 780/STAT-3 inhibitor co-treated samples were assessed by Student’s *t* test in GraphPad Prism 6. ***p < 0.001, **p < 0.01, *p < 0.05. Error bars represent the standard error of the mean (SEM) for independent cultures (*n* = 3). Proposed mechanism of PhIP-mediated CYP2E1 induction (**d**)

To further test the role of STAT3, MCF-7 cells were co-treated with the STAT3 inhibitor VIII 5, 15-diphenylporphyrin (25 µM) and PhIP for 24 h and CYP2E1 expression was determined by qPCR. Incubation with the STAT3 inhibitor completely abolished CYP2E1 mRNA upregulation by PhIP further supporting the involvement of STAT3 in its regulation (Fig. 4a).

Interestingly, PhIP increased CYP2E1 mRNA expression only in ER-α (+) MCF-7 cells (Fig. 3). PhIP has potent estrogenic activity mediated via ER-α (Lauber et al. 2004) and recently it has been shown that ER-α binds to STAT3 and JAK2 resulting in upregulation of JAK2-mediated STAT3 expression (Binai et al. 2010). We hypothesized that PhIP can activate ER-α to trigger the JAK/STAT pathway leading to the overexpression of CYP2E1. To investigate this, STAT3 and CYP2E1 mRNA expression was determined in MCF-7 cells following co-treatment with PhIP and ICI. ICI inhibited the PhIP-mediated STAT3 increase (Fig. 4b), and treatment with the ER inhibitor ICI significantly blocked CYP2E1 expression (Fig. 4c). The proposed mechanism for these interactions is summarised in Fig. 4d.

### Can CYP2E1 induction lead to ROS production by PhIP?

After establishing the induction of CYP2E1 mRNA by PhIP, we investigated whether CYP2E1 induction resulted in the generation of reactive oxygen species (ROS), since CYP2E1 enzyme is easily uncoupled leading to potent induction of ROS (Jimenez-Lopez and Cederbaum 2005), and ROS production may play an important role in tumor initiation and progression (Cerutti 1985; Slaga et al. 1981; Trush and Kensler 1991).

**Fig. 5:**
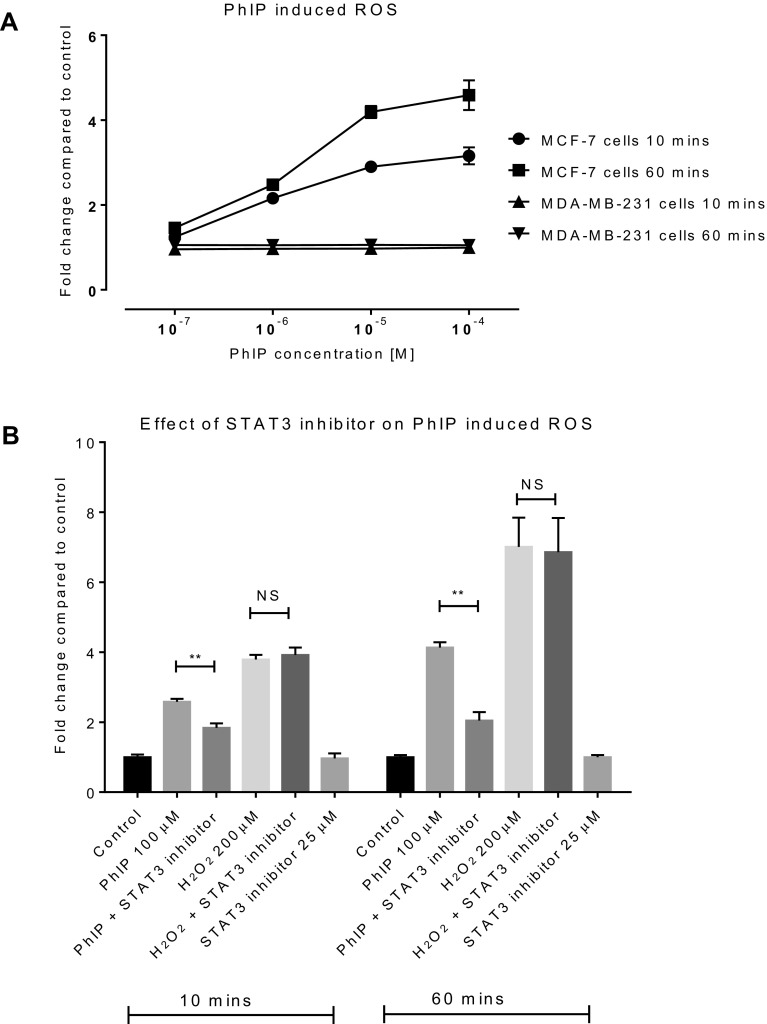
ROS generation following PhIP treatments in mammary cells: ROS production following PhIP treatments in MCF-7 and MDA-MB-231 cells (**a**) over 10 and 60 min. The effect of STAT-3 inhibition on ROS generation by PhIP was assessed in MCF-7 cells pretreated with PhIP with or without STAT-3 for 24 h and ROS (**b**) was monitored from 10 to 60 min. Statistically significant differences between PhIP vs. PhIP & STAT-3 co-treated samples were assessed by Student’s *t* test in GraphPad Prism 6. **p < 0.01, *NS* not significant. Error bars represent the standard error of the mean (SEM) for independent cultures (*n* = 3)

A dose-dependent increase in ROS levels was recorded following the treatment of MCF-7 cells (but not MDA-MB-231 cells) with PhIP (Fig. 5a). The effect was pronounced over the first 60 min then returned to control levels. This supports the hypothesis that CYP2E1 enzyme is involved in the generation of ROS in MCF-7 cells.

Since the STAT3 inhibitor completely blocked CYP2E1 mRNA expression, it was important to establish if STAT3 inhibition could modulate ROS generation. MCF-7 cells were treated with 100 µM PhIP (for 24 h) in the presence and absence of STAT3 inhibitor (25 µM) and ROS production was monitored. STAT-3 inhibitor significantly reduced ROS production by PhIP, providing evidence that CYP2E1 is a regulator of ROS production (Fig. 5b). Significantly, the addition of STAT3 inhibitor failed to attenuate the H_2_O_2_-induced ROS (Fig. 5b). However, it is important to note, although STAT3 inhibitor significantly inhibited ROS production, PhIP was still able to promote an oxidative stress response, implying that residual levels of CYP2E1 enzyme remained prone to uncoupling.

### Can co-exposure to ethanol and PhIP, potentiate DNA damage in mammary cells?

Our data show that PhIP can regulate CYP2E1-mediated oxidative stress, and the question arises whether these cellular affects could play a role in the initiation or progression of PhIP-induced mammary cancer. Interestingly, ethanol also upregulates oxidative stress via CYP2E1 enzyme (Jin et al. 2013), which suggests that co-exposure to PhIP and ethanol could potentiate DNA damage. As red meat and cigarette smoke (both a source of PhIP) and ethanol consumption are the most important modifiable lifestyle risk factors for breast cancer (Scoccianti et al. 2014b; Stein and Colditz 2004), we investigated if together these two lifestyle carcinogens can potentiate DNA damage in mammary cells.

### CYP2E1 upregulation by co-treatment of ethanol and PhIP

**Fig. 6:**
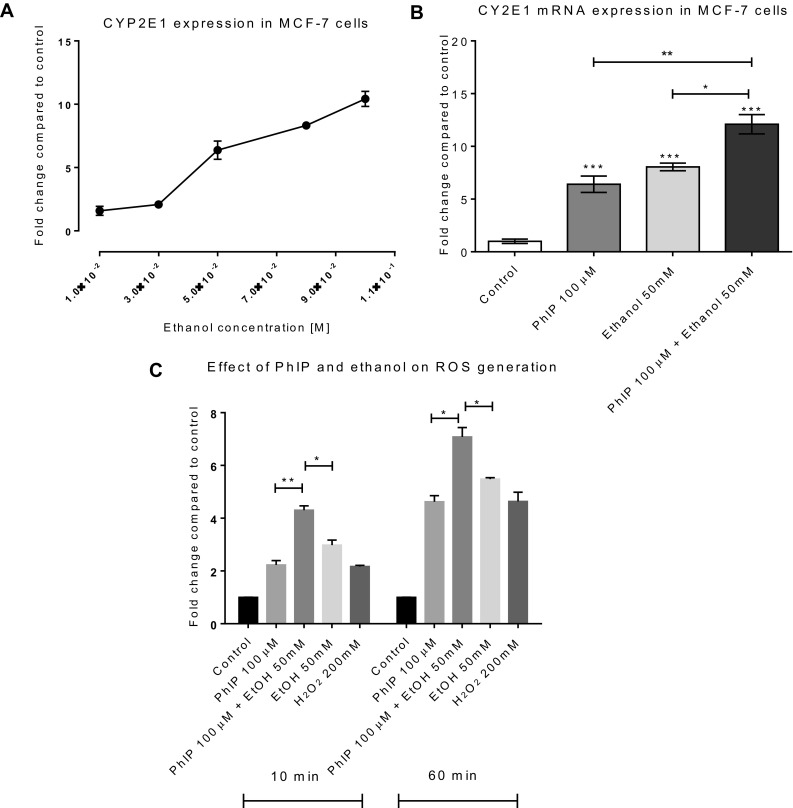
Increase in CYP2E1 expression and ROS generation by co-treatment of PhIP and ethanol: MCF-7 cells treated with ethanol (**a**) or PhIP 100 µM and ethanol 50 mM (**b**) for 24 h and CYP2E1 expression was determined by qPCR. Increase in ROS generation by co-treatment of PhIP and ethanol (**c**) in MCF-7 cells. Data is shown relative to control 0.1% DMSO. Statistically significant differences were calculated using one-way ANOVA with a Dunnett’s post test in GraphPad Prism 6, ***p < 0.0001–0.001, **p < 0.001–0.01. Error bars represent standard error of the mean (SEM) for independent cultures (*n* = 3)

CYP2E1 expression and activity are reported to play an important role in mammary carcinogenesis and provide a link between ethanol metabolism and progression of breast cancer (Leung et al. 2013). Published data show ethanol is capable of increasing CYP2E1, (Jin et al. 2013), (Roberts et al. 1995) and is known to increase ROS production (Bailey et al. 1999), (Sanchez-Alvarez et al. 2013), (Leon-Buitimea et al. 2012). Taking into consideration circulating ethanol levels after human alcohol consumption, CYP2E1 activity, and ROS production, we examined the effect of ethanol on MCF-7 cells. Since concentrations ranging from 10 to 100 mM correspond to the circulating levels of ethanol in blood following moderate to heavy drinking (Singletary et al. 2001), cells were treated with ethanol 10 to 100 mM and CYP2E1 mRNA expression was determined by qPCR. A dose-dependent increase in CYP2E1 expression was observed, which was significant at 50–100 mM (Fig. 6a).

We then examined the effect of co-treatment of 50 mM of ethanol and 100 µM of PhIP and a significant increase (*p* = 0.0144) in CYP2E1 mRNA expression was seen following co-treatment compared to ethanol or PhIP alone (Fig. 6b).

### Combined effect of ethanol and PhIP on ROS production

Although epidemiological studies provide convincing evidence about the involvement of ethanol in the increased incidence of breast cancer (Hamajima et al. 2002; Smith-Warner et al. 1998), the underlying molecular or cellular mechanisms are still not clear. The metabolism of ethanol by CYP2E1 and generation of ROS suggests a mechanism that can trigger genetic damage and/or maintain the tumor environment (Aye et al. 2004; Leung et al. 2013). Since our data show PhIP can induce oxidative stress (Fig. 5a), and recently it has been reported that ethanol can induce DNA damage *via* ROS generation (Guo et al. 2008; Kayani and Parry 2010) it was important to establish the effects of combinations of PhIP and ethanol on ROS formation.

MCF-7 cells were treated with ethanol (50 mM), PhIP (100 µM) or ethanol (50 mM) plus PhIP (100 µM) co-treatment and ROS production determined. Elevated intracellular ROS levels were observed by all treatments, with a statistically significant increase in ROS seen with co-treatment of PhIP plus ethanol compared to ethanol alone, at time points up to 60 min (Fig. 6c).

Overall, the ROS production (Fig. 6c) and increased CYP2E1 expression data (Fig. 6b) following co-treatment (PhIP + Ethanol) are consistent, suggesting increased CYP2E1 expression could be responsible for elevated ROS production.

### Can increase in ROS production lead to genotoxicity?

Increased oxidative stress can trigger or maintain the tumor environment and can also initiate tumorigenesis by inducing DNA damage (Cooke et al. 2003). Ethanol was generally considered non-genotoxic when examined in-vitro (Phillips and Jenkinson 2001), but recently it has been reported that ethanol can induce DNA damage via ROS generation (Guo et al. 2008; Kayani and Parry 2010). It is also pertinent that heterocyclic amines can be oxidatively activated by ROS to DNA damaging species (Banning et al. 1993). Since our data show simultaneous treatment with ethanol and PhIP-increased oxidative stress (Fig. 6c), and since ROS generation can induce oxidative DNA damage (Cooke et al. 2003), the genotoxicity of PhIP in the presence of ethanol was evaluated.

**Fig. 7:**
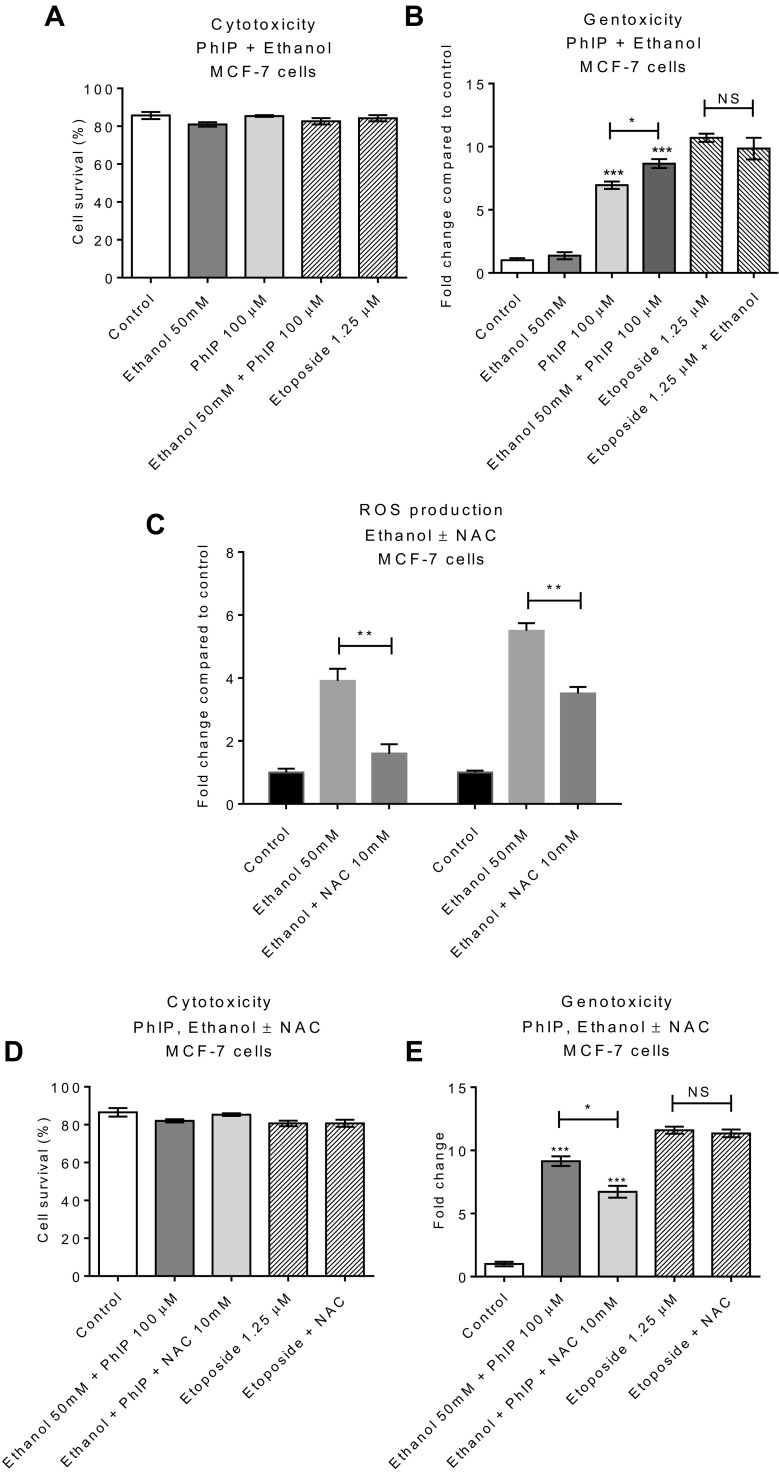
Increase in the genotoxicity of PhIP by ethanol: MCF-7 cells were treated with ethanol, PhIP or ethanol plus PhIP for 24 h, following treatment cells were allowed to recover for 48 h and then analyzed for cytotoxicity (**a**) and genotoxicity (**b**). micronucleus (MN) frequency per 1000 cells was measured following treatment (1000 cells/slide and two slides per culture). Etoposide was used as the positive control. Ethanol-induced ROS was inhibited by the addition of *N*-acetyl cysteine (NAC) (**c**). Inclusion of NAC in incubations did not affect cytotoxicity (**d**), but ethanol plus PhIP-mediated genotoxicity was attenuated (**e**). Data are shown relative to control 0.1% DMSO. Statistically significant differences for treatments vs. control were calculated using one-way ANOVA with a Dunnett’s post test in GraphPad Prism 6, ***p < 0.001, **p < 0.01. Error bars represent standard error of the mean (SEM) for independent cultures (*n* = 3). Statistically significant difference between the indicated treatments was determined by Student’s *t* test GraphPad Prism 6, **p < 0.01, *p < 0.05, *NS* not significant

MCF-7 cells were treated with PhIP (100 µM), ethanol (50 mM) or PhIP plus ethanol for 24 h, cells were harvested after 48 h and analysed for cytotoxicity and genotoxicity (MN induction). A minimal decrease in cell survival was seen with treatments (Fig. 7a). Treatment with ethanol did not significantly induce MN, consistent with previous reports (Phillips and Jenkinson 2001) (Fig. 7b). However, a significant increase in the genotoxicity was seen following co-treatment of ethanol with PhIP compared to PhIP alone (Fig. 7b), consistent with the notion that high levels of ROS generation can contribute to the DNA damage. No change in the genotoxicity of etoposide by the addition of ethanol was observed (Fig. 7b). This is entirely consistent with the genotoxic action of etoposide (topoisomerase inhibition), which is independent of ROS involvement. These data indicate that ethanol potentiated the effect of the other ROS-generating agent (PhIP), leading to increased oxidative DNA damage. We suggest that the increased level of DNA damage is linked to the PhIP-induced expression of CYP2E1, which in turn potentiates activation of ethanol to DNA-damaging ROS. We further suggest that the generation of ROS could also activate PhIP by one electron oxidation to DNA damaging species. This mechanism proposes that ethanol can indirectly increase the genotoxicity of other chemicals (e.g., PhIP in meat) that are routinely consumed with alcohol.

To further confirm that the increase in the genotoxicity of PhIP in the presence of ethanol is due to ROS production, we attempted to block the production of ROS and investigate the effect on genotoxicity. *N*-acetyl-cysteine (NAC) is a ROS scavenger and shown previously to abolish accumulation of ROS at 10 mM concentration in MCF-7 cells (Li et al. 2013; Martinez-Outschoorn et al. 2010). Cells were treated with 50 mM of ethanol and 50 mM of ethanol plus 10 mM of NAC and examined for ROS generation; a clear decrease in ROS production was seen in the presence of NAC (Fig. 7c). The effect of treatment of MCF-7 cells with ethanol (50 mM) and PhIP (100 µM) in the presence of NAC (10 mM) was investigated and the cytotoxicity and genotoxicity (MN induction) examined. Treatment with NAC did not affect cell survival (Fig. 7d). Addition of NAC decreased the genotoxicity of PhIP plus ethanol significantly (*p* = 0.01) (Fig. 7e), providing evidence that by blocking ROS production, the genotoxicity is decreased. Importantly, NAC had little effect on the genotoxicity of positive control etoposide (Fig. 7e), consistent with etoposide’s different mechanism of DNA damage (topoisomerase inhibition).

## Discussion

The cooked food-derived mutagen PhIP is a genotoxic rat procarcinogen. PhIP is metabolically activated by the CYP1 family of enzymes (particularly CYP1A2) to *N*-hydroxy-PhIP (Boobis et al. 1994; Zhao et al. 1994). Subsequent esterification of the *N*-hydroxy metabolite produces unstable products that spontaneously form nitrenium ions that react with DNA to form covalent adducts that, unless repaired, may lead to mutagenesis (Buonarati et al. 1990; Chevereau et al. 2017; Crosbie et al. 2000; Langouet et al. 2002; Turesky et al. 2002). Generally, metabolic activation of procarcinogens occurs in the liver where there is an abundance of CYP enzymes expressed (Guengerich 2017). Indeed the highest levels of CYP1A2 expression in the rat occurs in the liver, yet PhIP is not a hepatic carcinogen in the rat. PhIP can also act as a substrate for CYP1A1 and CYP1B1 enzymes, which can convert it to the genotoxic *N*-hydroxy metabolite (Boobis et al. 1994; Zhao et al. 1994). Both CYP1A1 and CYP1B1 are expressed extra-hepatic and are under regulatory control of the Ah receptor (Hankinson 2016). Dietary exposure of rats to PhIP results in tumours of the colon, mammary gland and the prostate (Ito et al. 1991; Shirai et al. 1997), which aligns with diet-associated cancers in humans (Gooderham et al. 2007, 1996). PhIP has, therefore, been studied as a potential model dietary carcinogen. Whilst the CYP1 family-mediated activation and esterification of PhIP is well understood as the genotoxic mode of action, the tissue specificity of PhIP’s carcinogenicity is not so well understood.

We have previously reported that PhIP has hormonal-like activity being able to act as an ER ligand with specificity for ER-α (Lauber et al. 2004; Lauber and Gooderham 2007, 2011). This latter property of PhIP is known to promote human mammary cell proliferation (Lauber et al. 2004; Lauber and Gooderham 2007), cell migration and invasion (Lauber and Gooderham 2011). All of these events are key to the development of cancer. Whilst undoubtedly a genotoxic carcinogen, the additional hormone-like properties of PhIP are consistent with its tissue-selective tumourigenicity in rats (breast, prostate and colon) (Ito et al. 1991; Shirai et al. 1997). Importantly, PhIP’s specificity for ER-α appears to be a key activity in its ability to drive the cancer process. This ER-α specificity is further emphasized in the present study.

### The role of ER-α in the genotoxicity of PhIP in MCF-7 cells

The current study has shown that PhIP is a powerful inducer of genotoxicity in MCF-7 cells but less effective in MDA-MB-231 cells. Previously, Fischer and colleagues demonstrated that estradiol can induce micronuclei in MCF-7 but not in MDA-MB-231 cells (Fischer et al. 2001); similar responses have been reported for the estrogenic compound bisphenol A (Iso et al. 2006), suggesting the selective genotoxicity of estrogenic compounds in hormone responsive cell line (MCF-7). In line with this, here we show the genotoxicity of PhIP is inhibited by treatment with ER antagonist ICI182780 in MCF-7, but is unaffected in MDA-MB-231 cells.

In the present study, we link PhIP-mediated induction of CYP1B1 to selective genotoxicity in MCF-7 cells, compared to MDA-MB-231 cells. MDA-MB-231 cells only express ER-β and MCF-7 cells express both ER-α and ER-β. PhIP displays its estrogenic effects selectively via ER-α (Lauber et al. 2004), thus PhIP-mediated induction of CYP1B1 in MCF-7 cells is likely through the same mechanism of ER-α-ERE (estrogen responsive element) interaction. Inhibition of PhIP-mediated CYP1B1 mRNA induction by ER antagonist (ICI 182,780), strongly supports the role of ER receptor in CYP1B1 gene regulation in MCF-7 cells. Consistent with this, Tsuchiya et al. (2004) and others showed that estradiol could induce CYP1B1 expression in ER-α-positive MCF-7 cells but not in ER-α-negative MDA-MB-345 cells, by direct interaction of liganded-ER-α with ERE on the CYP1B1 gene (Tsuchiya et al. 2004) (Han et al. 2010; Mookherjee et al. 2012). Estradiol has the ability to induce tumorigenic potential in benign MCF-10F cells and knock-down of ER-α receptor can delay the onset of tumors in rats (Santen et al. 2009), suggesting that ER-α activation by xenoestrogens such as PhIP can have similar implications as estradiol.

### CYP2E1-mediated ROS generation by PhIP

CYP2E1 is primarily expressed in liver but has been detected in other tissues such as breast, brain, kidney and lungs (Leung et al. 2013). Most studies on CYP2E1 are in relation to liver diseases (Liu et al. 2005) including the metabolism of ethanol (Leon-Buitimea et al. 2012). Clinical studies have shown that CYP2E1 is highly expressed in breast tumors compared to normal breast tissue (Kapucuoglu et al. 2003). The present study presents a novel mechanism by which the dietary carcinogen PhIP can upregulate CYP2E1 expression, which consequently promotes oxidative stress in breast carcinoma cells.

Interestingly, PhIP-induced CYP2E1 mRNA only in ER-α-positive MCF-7 cells and not in the ER-α-negative MDA-MB-231 cells. The mechanism appears to involve activity of ER-α (Lauber et al. 2004) leading to upregulation of JAK/STAT3 pathway thereby inducing CYP2E1. In support of this, it has been reported that in MCF-7 cells ER-α can bind to STAT3/JAK2 leading to their upregulation (Binai et al. 2010). Importantly, CYP2E1 promoter region has multiple binding sites for STAT that are involved in CYP2E1 upregulation (Patel et al. 2014). In future studies, it will be important to confirm the activation of STAT3 in this pathway by investigating site-specific protein phosphorylation.

Our data also show that PhIP-mediated CYP2E1 induction leads to generation of oxidative stress that can be attenuated by STAT3 inhibitor, suggesting involvement of both STAT3 and CYP2E1 in this process. However, although STAT3 inhibitor reduced the PhIP-mediated production of ROS, it was not completely eliminated, suggesting that PhIP might be generating ROS by more than one mechanism. Consistent with this, Lauber et al. (Lauber et al. 2004; Lauber and Gooderham 2007) showed that PhIP activates the ERK/MAPK pathway and Chaudhary et al. showed that PhIP can generate ROS in MCF-10A cells via extracellular signal-regulated kinase (ERK) pathway activation (Choudhary et al. 2012).

Estradiol can induce DNA damage via ROS generation in MCF-7 but not in MDA-MB-231 cells (Mobley and Brueggemeier 2004), thus suggesting the role of ER in oxidative DNA damage. Interestingly, Liehr et al. (2001) showed that estrogen can induce DNA damage directly by forming reactive metabolites or indirectly by promoting reactive oxygen species (ROS) and redox cycling. These observations are consistent with data presented here and emphasise that estrogenic agents like PhIP can generate oxidative stress leading to DNA damage.

Both in human and experimental studies, DNA damage by ROS production has been widely proposed as a major cause of cancer initiation and promotion (Loft and Poulsen 1996; Poulsen et al. 1998; Waris and Ahsan 2006). Mutations induced by ROS are primarily transversions of Guanine to Thymine (Du et al. 1994; Higinbotham et al. 1992), and this is consistent with the preponderance of mutations induced by PhIP (Lynch et al. 1998; Yadollahi-Farsani et al. 1996). The ability of PhIP to induce CYP2E1 has importance beyond ROS production since CYP2E1 also metabolizes low-molecular weight molecules such as ethanol, acetaminophen and procarcinogens like nitrosamines and azo compounds (Gonzalez 2005).

### Consumption of ethanol and red meat and breast cancer

**Fig. 8:**
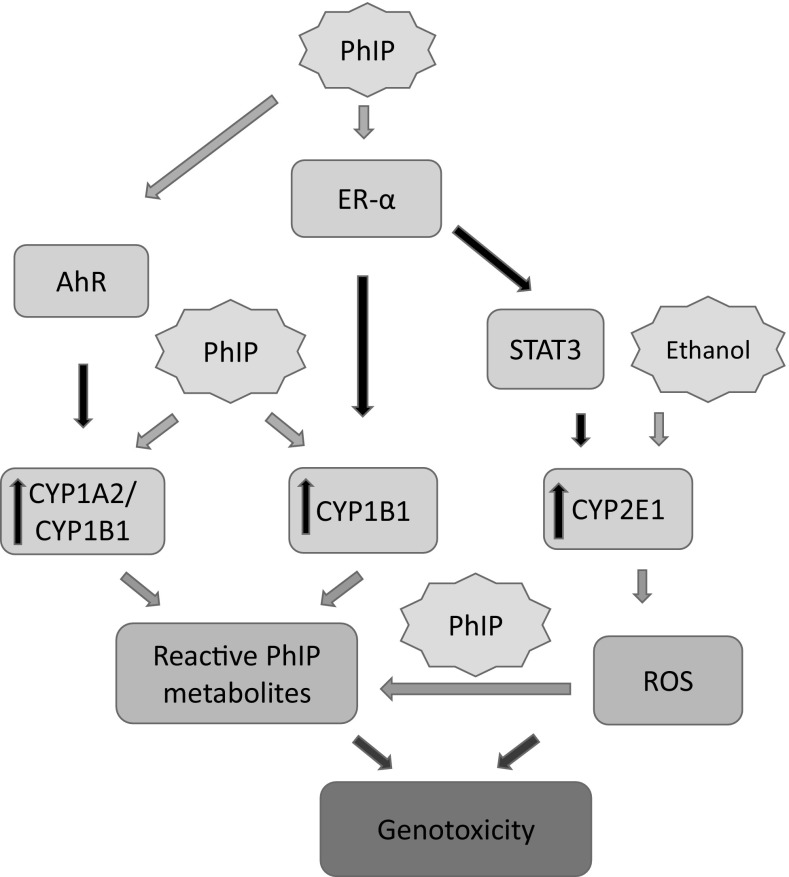
Proposed mechanism by which PhIP and ethanol induces genotoxicity in MCF-7 cells. PhIP can activate the AhR leading to upregulation of CYP1A2 /1B1 and increased metabolism of PhIP to genotoxic metabolites. PhIP can also upregulate CYP1B1 and CYP2E1 via ER-α through mir27b and STAT3, respectively. Additionally, ethanol can upregulate CYP2E1 expression. Collectively, these gene expression changes can lead to increased metabolic activation of carcinogenic substrates and higher levels of oxidative stress resulting in genotoxicity

Epidemiological studies provide a well-established link between lifestyle factors such as cooked meat and alcohol consumption and the development of breast cancer, yet the mechanistic basis of these associations is not well understood (Leon-Buitimea et al. 2012; Singletary 1997). Recreational consumption of ethanol in women can rapidly lead to mM plasma concentrations; such consumption can be frequent and addictive. Here, we propose a mechanism that links DNA damage, CYP1B1, CYP2E1, ROS and ER-α with the metabolism of ethanol and the cooked meat-derived procarcinogen PhIP (Fig. 8).

Following ethanol or PhIP treatment of human mammary cells, an increase in intracellular ROS levels was observed that was dependent on a functional ER-α; this is in agreement with published observations using the breast carcinoma cell line MCF-10A (Leon-Buitimea et al. 2012) (Choudhary et al. 2012). Importantly, our study also shows that co-treatments of ethanol and PhIP can increase the expression of CYP2E1 that in turn can generate oxidative stress leading to increased DNA damage. Additionally, it has been reported by ourselves and others that heterocyclic amines including PhIP are activated by one electron oxidation mechanisms such as with ROS, peroxidase and lactoperoxidase to DNA-damaging species (Banning et al. 1993; Gorlewska-Roberts et al. 2004; Moonen et al. 2002).

Consumption of alcohol has been shown to increase the level of endogenous estrogens (Scoccianti et al. 2014a) and PhIP is estrogenic in nature. PhIP can induce the expression of CYP2E1 resulting in increased metabolism of alcohol and subsequent generation of ROS. PhIP is a powerful genotoxicant and mammary carcinogen and ROS is genotoxic and tumour promoting. It has been reported that ethanol can increase the invasion of breast cancer cells by modulating matrix metalloproteinase-2 (MMP-2) (Aye et al. 2004), while PhIP also increased the invasiveness of breast cancer cells (Lauber and Gooderham 2011), therefore, together these two compounds can potentially support and promote the progression of mammary cancer.

These activities, summarised in Fig. 8, provide a basis for explaining the observation that co-exposure to PhIP and ethanol can lead to the initiation and promotion of breast cancer. Both red meat and alcohol co-consumption is common in the western world, and both are associated with the incidence of breast cancer. This current study offers a mechanistic basis for this association.
